# Supplementary material for: Predicting visual function by interpreting a neuronal wiring diagram
Source: Nature. 2024 Oct 2;634(8032):113–23. doi: 10.1038/s41586-024-07953-5 (PMC11446822; doi:10.1038/s41586-024-07953-5)
Supplement: Supplementary file 6 — CRF and ERF predictions for individual TmY4 and TmY9 cells. Analogous to Supplementary Data 3, but for TmY target types. Shown are the top four monosynaptic pathways, the strongest pathway passing through each of the top ten intermediary types (ranking from Extended Data Fig. 7), and the trisynaptic pathway Tm1–TmY–Dm3–TmY (see the section entitled Prediction of spatial normalization). [file 41586_2024_7953_MOESM6_ESM.zip › DataS4/TmY9qΓèÑ/720575940620743883.pdf]

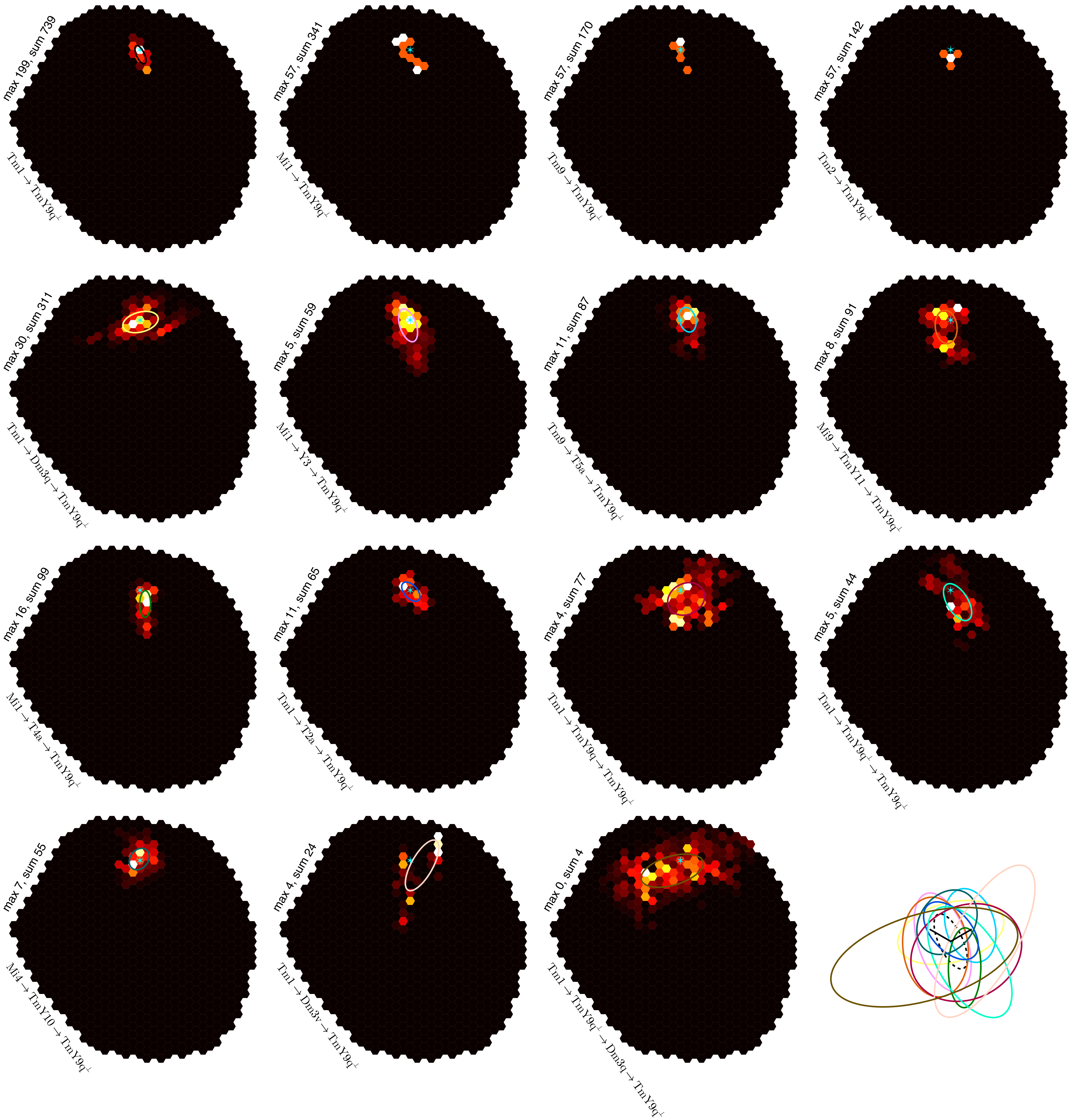
max 199, sum 739
Tm1 → TmY9q⊥
max 57, sum 341
Mi1 → TmY9q⊥
max 57, sum 170
Tm9 → TmY9q⊥
max 57, sum 142
Tm2 → TmY9q⊥
max 30, sum 311
Tm1 → Dm3q → TmY9q⊥
max 5, sum 59
Mi1 → Y3 → TmY9q⊥
max 11, sum 87
Tm9 → T5a → TmY9q⊥
max 8, sum 91
Mi9 → TmY11 → TmY9q⊥
max 16, sum 99
Mi1 → T4a → TmY9q⊥
max 11, sum 65
Tm1 → T2a → TmY9q⊥
max 4, sum 77
Tm1 → TmY9q → TmY9q⊥
max 5, sum 44
Tm1 → TmY9q⊥ → TmY9q⊥
max 7, sum 55
Mi4 → TmY10 → TmY9q⊥
max 4, sum 24
Tm1 → Dm3v → TmY9q⊥
max 0, sum 4
Tm1 → TmY9q⊥ → Dm3q → TmY9q⊥
